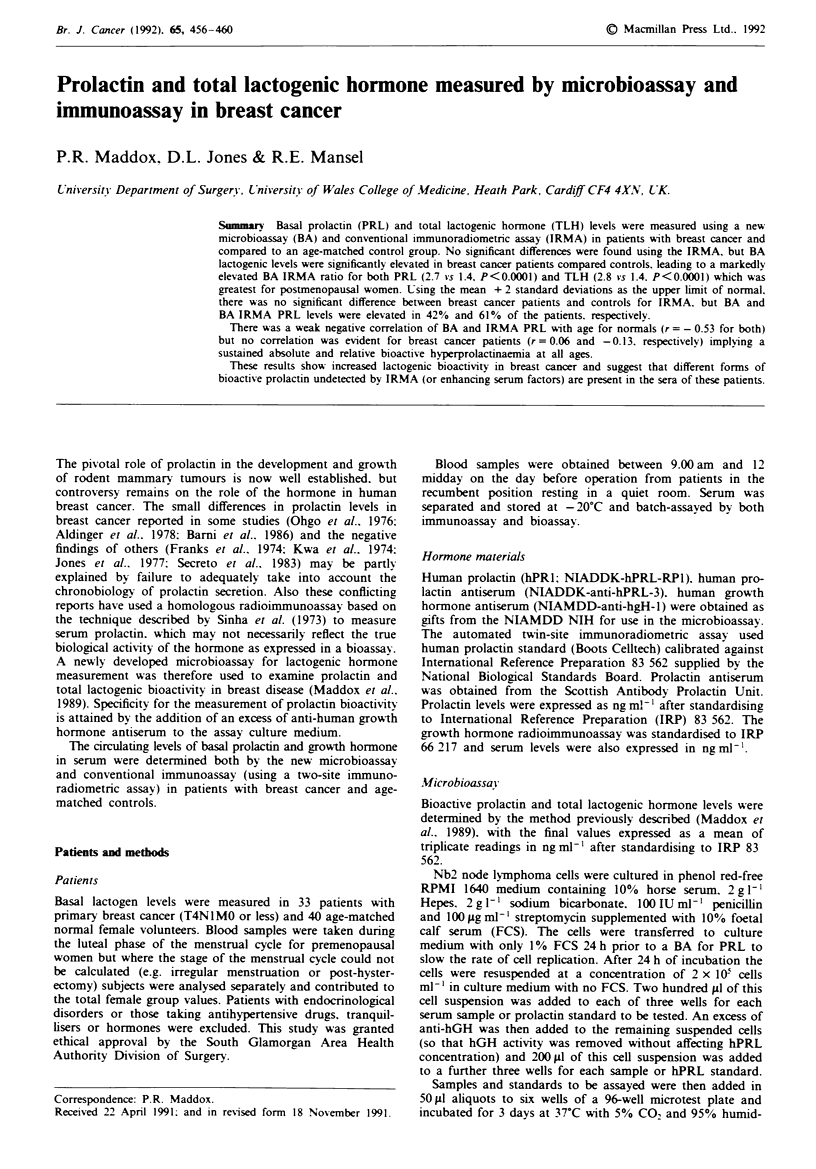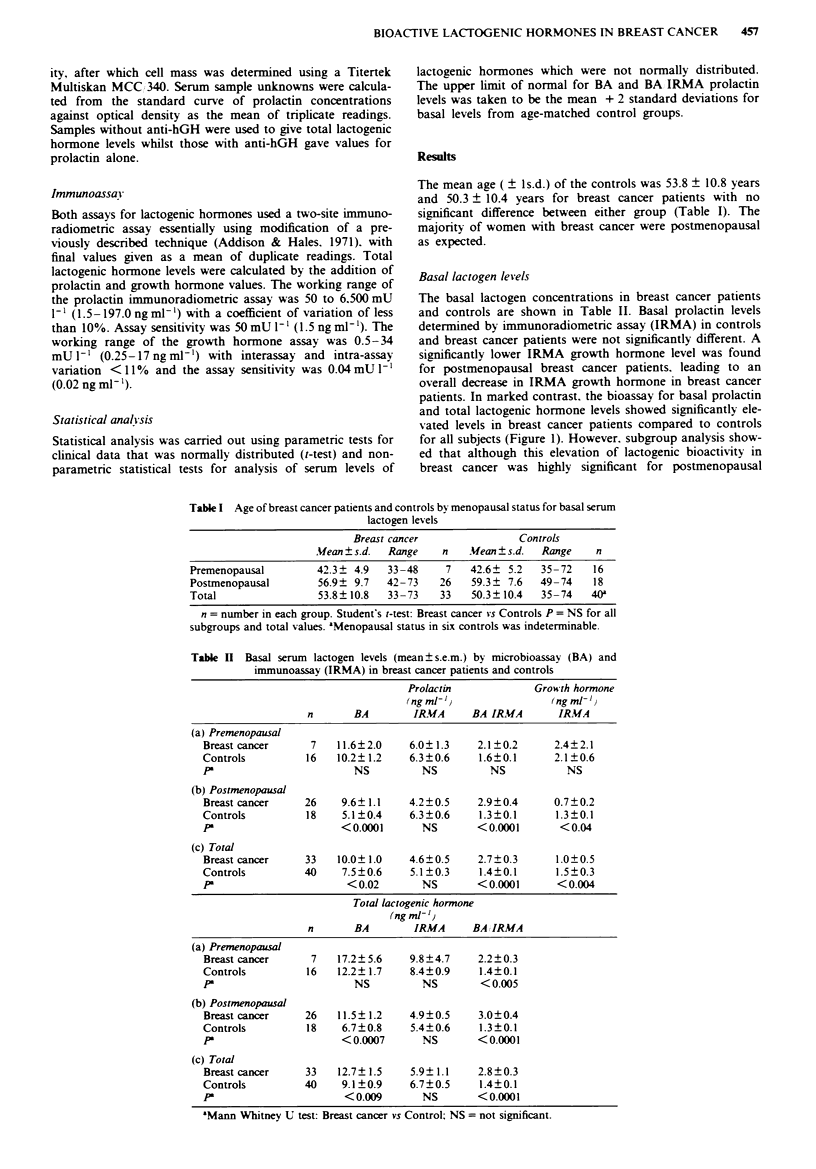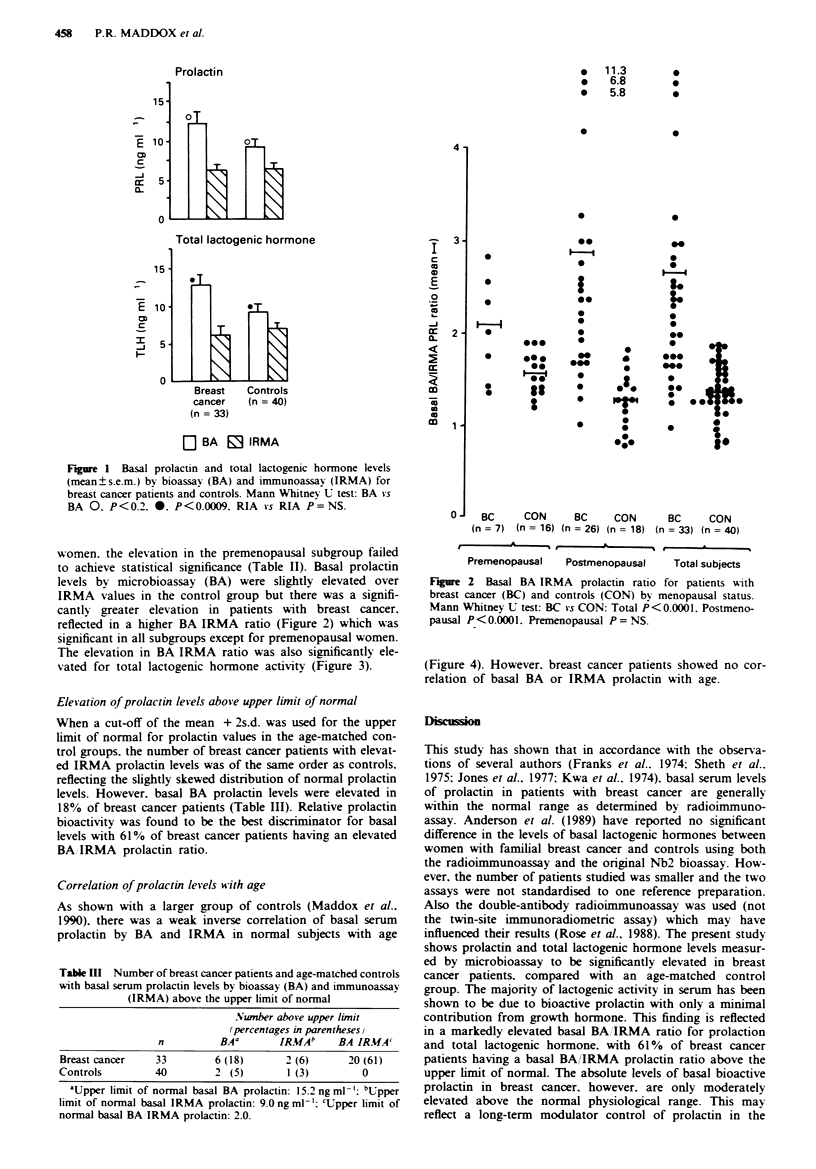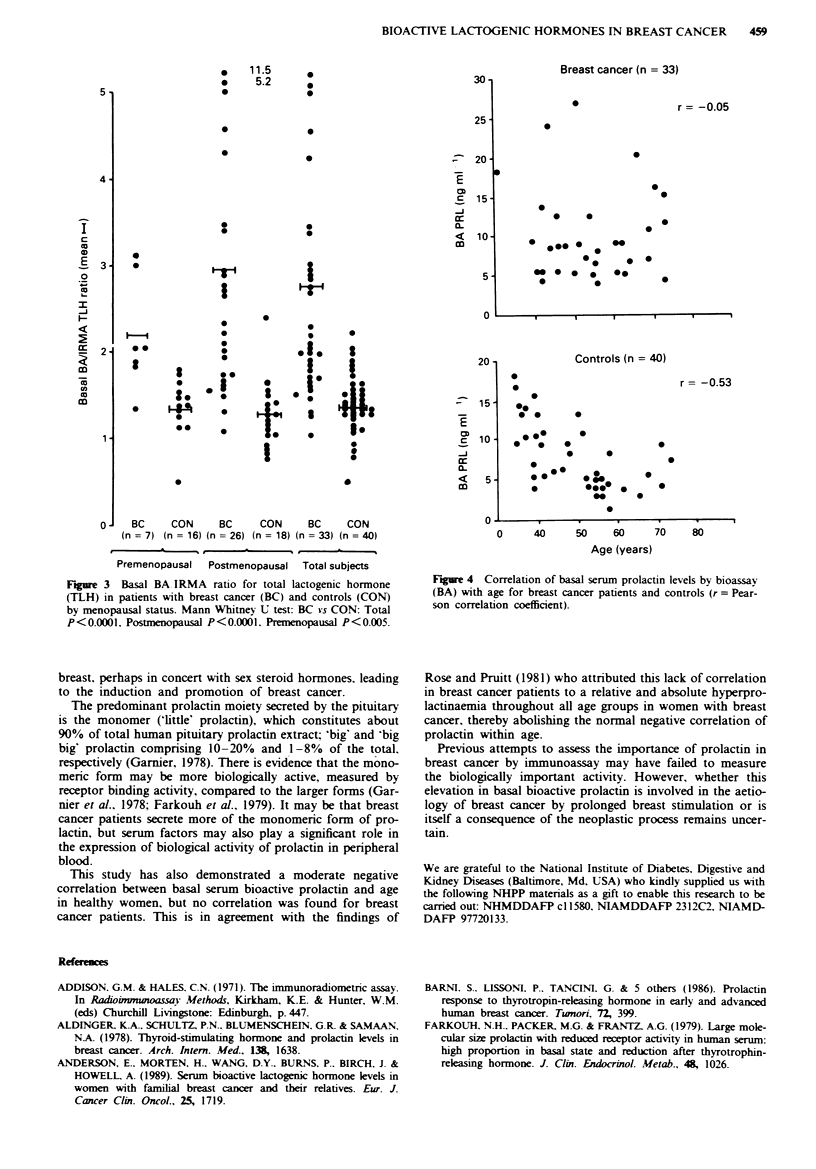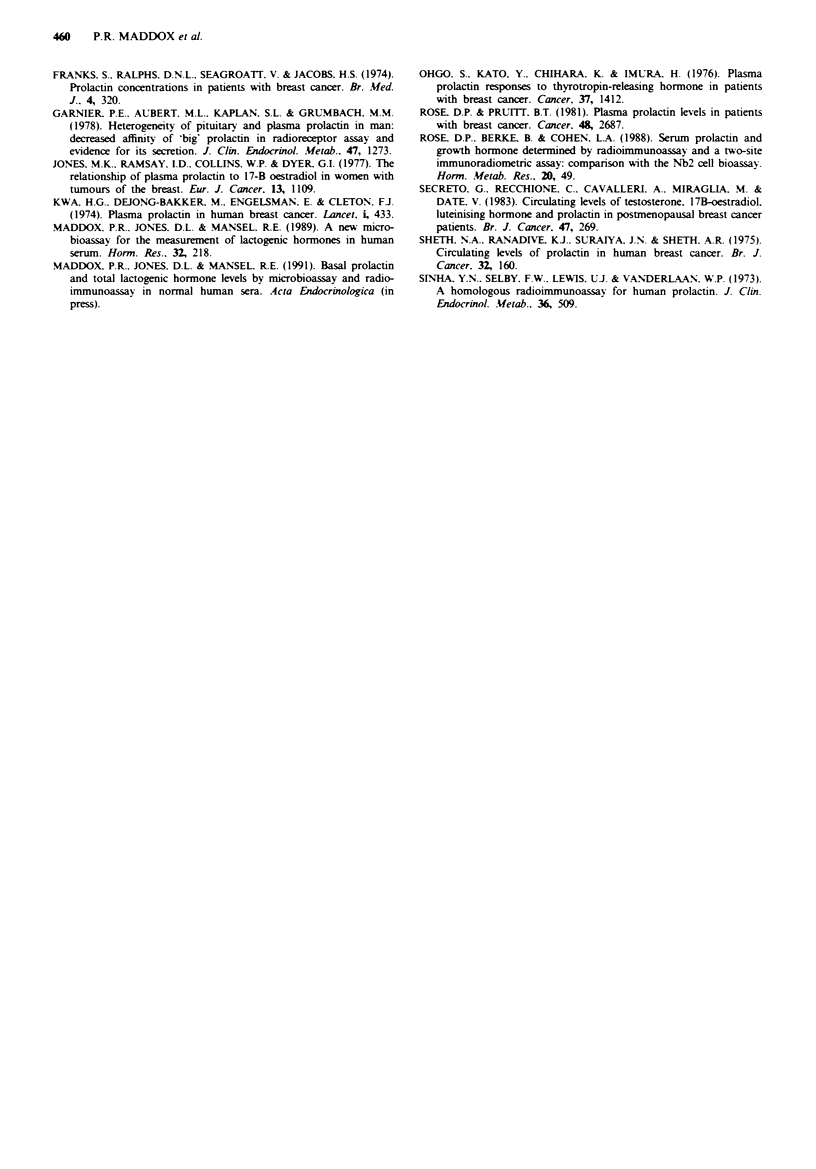# Prolactin and total lactogenic hormone measured by microbioassay and immunoassay in breast cancer.

**DOI:** 10.1038/bjc.1992.92

**Published:** 1992-03

**Authors:** P. R. Maddox, D. L. Jones, R. E. Mansel

**Affiliations:** University Department of Surgery, University of Wales College of Medicine, Heath Park, Cardiff, UK.

## Abstract

Basal prolactin (PRL) and total lactogenic hormone (TLH) levels were measured using a new microbioassay (BA) and conventional immunoradiometric assay (IRMA) in patients with breast cancer and compared to an age-matched control group. No significant differences were found using the IRMA, but BA lactogenic levels were significantly elevated in breast cancer patients compared controls, leading to a markedly elevated BA/IRMA ratio for both PRL (2.7 vs 1.4, P less than 0.0001) and TLH (2.8 vs 1.4, P less than 0.0001) which was greatest for postmenopausal women. Using the mean +2 standard deviations as the upper limit of normal, there was no significant difference between breast cancer patients and controls for IRMA, but BA and BA/IRMA PRL levels were elevated in 42% and 61% of the patients, respectively. There was a weak negative correlation of BA and IRMA PRL with age for normals (r = -0.53 for both) but no correlation was evident for breast cancer patients (r = 0.06 and -0.13, respectively) implying a sustained absolute and relative bioactive hyperprolactinaemia at all ages. These results show increased lactogenic bioactivity in breast cancer and suggest that different forms of bioactive prolactin undetected by IRMA (or enhancing serum factors) are present in the sera of these patients.


Br. J. Cancer (1992), 65, 456-460 © Macmillan Press Ltd., 1992

### Prolactin and total lactogenic hormone measured by microbioassay and immunoassay in breast cancer

P.R. Maddox, D.L. Jones & R.E. Mansel

University Department of Surgery, University of Wales College of Medicine, Heath Park, Cardiff CF4 4XN, UK.

**Summary** Basal prolactin (PRL) and total lactogenic hormone (TLH) levels were measured using a new microbioassay (BA) and conventional immunoradiometric assay (IRMA) in patients with breast cancer and compared to an age-matched control group. No significant differences were found using the IRMA, but BA lactogenic levels were significantly elevated in breast cancer patients compared controls, leading to a markedly elevated BA IRMA ratio for both PRL (2.7 *vs* 1.4, $P<0.0001$) and TLH (2.8 *vs* 1.4, $P<0.0001$) which was greatest for postmenopausal women. Using the mean +2 standard deviations as the upper limit of normal, there was no significant difference between breast cancer patients and controls for IRMA, but BA and BA IRMA PRL levels were elevated in 42% and 61% of the patients, respectively.

There was a weak negative correlation of BA and IRMA PRL with age for normals ($r = -0.53$ for both) but no correlation was evident for breast cancer patients ($r = 0.06$ and $-0.13$, respectively) implying a sustained absolute and relative bioactive hyperprolactinaemia at all ages.

These results show increased lactogenic bioactivity in breast cancer and suggest that different forms of bioactive prolactin undetected by IRMA (or enhancing serum factors) are present in the sera of these patients.

The pivotal role of prolactin in the development and growth of rodent mammary tumours is now well established, but controversy remains on the role of the hormone in human breast cancer. The small differences in prolactin levels in breast cancer reported in some studies (Ohgo *et al.*, 1976; Aldinger *et al.*, 1978; Barni *et al.*, 1986) and the negative findings of others (Franks *et al.*, 1974; Kwa *et al.*, 1974; Jones *et al.*, 1977; Secreto *et al.*, 1983) may be partly explained by failure to adequately take into account the chronobiology of prolactin secretion. Also these conflicting reports have used a homologous radioimmunoassay based on the technique described by Sinha *et al.* (1973) to measure serum prolactin, which may not necessarily reflect the true biological activity of the hormone as expressed in a bioassay. A newly developed microbioassay for lactogenic hormone measurement was therefore used to examine prolactin and total lactogenic bioactivity in breast disease (Maddox *et al.*, 1989). Specificity for the measurement of prolactin bioactivity is attained by the addition of an excess of anti-human growth hormone antiserum to the assay culture medium.

The circulating levels of basal prolactin and growth hormone in serum were determined both by the new microbioassay and conventional immunoassay (using a two-site immunoradiometric assay) in patients with breast cancer and age-matched controls.

### Patients and methods

### Patients

Basal lactogen levels were measured in 33 patients with primary breast cancer (T4N1M0 or less) and 40 age-matched normal female volunteers. Blood samples were taken during the luteal phase of the menstrual cycle for premenopausal women but where the stage of the menstrual cycle could not be calculated (e.g. irregular menstruation or post-hysterectomy) subjects were analysed separately and contributed to the total female group values. Patients with endocrinological disorders or those taking antihypertensive drugs, tranquillisers or hormones were excluded. This study was granted ethical approval by the South Glamorgan Area Health Authority Division of Surgery.

Correspondence: P.R. Maddox.
Received 22 April 1991; and in revised form 18 November 1991.

Blood samples were obtained between 9.00 am and 12 midday on the day before operation from patients in the recumbent position resting in a quiet room. Serum was separated and stored at −20°C and batch-assayed by both immunoassay and bioassay.

### Hormone materials

Human prolactin (hPR1; NIADDK-hPRL-RP1), human prolactin antiserum (NIADDK-anti-hPRL-3), human growth hormone antiserum (NIAMDD-anti-hgH-1) were obtained as gifts from the NIAMDD NIH for use in the microbioassay. The automated twin-site immunoradiometric assay used human prolactin standard (Boots Celltech) calibrated against International Reference Preparation 83 562 supplied by the National Biological Standards Board. Prolactin antiserum was obtained from the Scottish Antibody Prolactin Unit. Prolactin levels were expressed as ng $ml^{-1}$ after standardising to International Reference Preparation (IRP) 83 562. The growth hormone radioimmunoassay was standardised to IRP 66 217 and serum levels were also expressed in ng $ml^{-1}$.

### Microbioassay

Bioactive prolactin and total lactogenic hormone levels were determined by the method previously described (Maddox *et al.*, 1989), with the final values expressed as a mean of triplicate readings in ng $ml^{-1}$ after standardising to IRP 83 562.

Nb2 node lymphoma cells were cultured in phenol red-free RPMI 1640 medium containing 10% horse serum, 2 g $l^{-1}$ Hepes, 2 g $l^{-1}$ sodium bicarbonate, 100 IU $ml^{-1}$ penicillin and 100 $\mu g$ $ml^{-1}$ streptomycin supplemented with 10% foetal calf serum (FCS). The cells were transferred to culture medium with only 1% FCS 24 h prior to a BA for PRL to slow the rate of cell replication. After 24 h of incubation the cells were resuspended at a concentration of $2 \times 10^5$ cells $ml^{-1}$ in culture medium with no FCS. Two hundred $\mu l$ of this cell suspension was added to each of three wells for each serum sample or prolactin standard to be tested. An excess of anti-hGH was then added to the remaining suspended cells (so that hGH activity was removed without affecting hPRL concentration) and 200 $\mu l$ of this cell suspension was added to a further three wells for each sample or hPRL standard.

Samples and standards to be assayed were then added in 50 $\mu l$ aliquots to six wells of a 96-well microtest plate and incubated for 3 days at 37°C with 5% $CO_2$ and 95% humidity, after which cell mass was determined using a Titertek Multiskan MCC/340. Serum sample unknowns were calculated from the standard curve of prolactin concentrations against optical density as the mean of triplicate readings. Samples without anti-hGH were used to give total lactogenic hormone levels whilst those with anti-hGH gave values for prolactin alone.

*Immunoassay*

Both assays for lactogenic hormones used a two-site immunoradiometric assay essentially using modification of a previously described technique (Addison & Hales, 1971), with final values given as a mean of duplicate readings. Total lactogenic hormone levels were calculated by the addition of prolactin and growth hormone values. The working range of the prolactin immunoradiometric assay was 50 to 6,500 mU $l^{-1}$ (1.5–197.0 ng $ml^{-1}$) with a coefficient of variation of less than 10%. Assay sensitivity was 50 mU $l^{-1}$ (1.5 ng $ml^{-1}$). The working range of the growth hormone assay was 0.5–34 mU $l^{-1}$ (0.25–17 ng $ml^{-1}$) with interassay and intra-assay variation <11% and the assay sensitivity was 0.04 mU $l^{-1}$ (0.02 ng $ml^{-1}$).

*Statistical analysis*

Statistical analysis was carried out using parametric tests for clinical data that was normally distributed (*t*-test) and non-parametric statistical tests for analysis of serum levels of lactogenic hormones which were not normally distributed. The upper limit of normal for BA and BA/IRMA prolactin levels was taken to be the mean + 2 standard deviations for basal levels from age-matched control groups.

### Results

The mean age (± 1s.d.) of the controls was 53.8 ± 10.8 years and 50.3 ± 10.4 years for breast cancer patients with no significant difference between either group (Table I). The majority of women with breast cancer were postmenopausal as expected.

*Basal lactogen levels*

The basal lactogen concentrations in breast cancer patients and controls are shown in Table II. Basal prolactin levels determined by immunoradiometric assay (IRMA) in controls and breast cancer patients were not significantly different. A significantly lower IRMA growth hormone level was found for postmenopausal breast cancer patients, leading to an overall decrease in IRMA growth hormone in breast cancer patients. In marked contrast, the bioassay for basal prolactin and total lactogenic hormone levels showed significantly elevated levels in breast cancer patients compared to controls for all subjects (Figure 1). However, subgroup analysis showed that although this elevation of lactogenic bioactivity in breast cancer was highly significant for postmenopausal

**Table I** Age of breast cancer patients and controls by menopausal status for basal serum lactogen levels

| | *Breast cancer* | | | *Controls* | | |
|---|---|---|---|---|---|---|
| | *Mean ± s.d.* | *Range* | *n* | *Mean ± s.d.* | *Range* | *n* |
| Premenopausal | 42.3 ± 4.9 | 33–48 | 7 | 42.6 ± 5.2 | 35–72 | 16 |
| Postmenopausal | 56.9 ± 9.7 | 42–73 | 26 | 59.3 ± 7.6 | 49–74 | 18 |
| Total | 53.8 ± 10.8 | 33–73 | 33 | 50.3 ± 10.4 | 35–74 | 40[a] |

*n* = number in each group. Student's *t*-test: Breast cancer *vs* Controls *P* = NS for all subgroups and total values. [a]Menopausal status in six controls was indeterminable.

**Table II** Basal serum lactogen levels (mean ± s.e.m.) by microbioassay (BA) and immunoassay (IRMA) in breast cancer patients and controls

| | *n* | *Prolactin (ng ml⁻¹)* BA | *Prolactin (ng ml⁻¹)* IRMA | *BA/IRMA* | *Growth hormone (ng ml⁻¹)* IRMA |
|---|---|---|---|---|---|
| (a) *Premenopausal* | | | | | |
| Breast cancer | 7 | 11.6 ± 2.0 | 6.0 ± 1.3 | 2.1 ± 0.2 | 2.4 ± 2.1 |
| Controls | 16 | 10.2 ± 1.2 | 6.3 ± 0.6 | 1.6 ± 0.1 | 2.1 ± 0.6 |
| *P*[a] | | NS | NS | NS | NS |
| (b) *Postmenopausal* | | | | | |
| Breast cancer | 26 | 9.6 ± 1.1 | 4.2 ± 0.5 | 2.9 ± 0.4 | 0.7 ± 0.2 |
| Controls | 18 | 5.1 ± 0.4 | 6.3 ± 0.6 | 1.3 ± 0.1 | 1.3 ± 0.1 |
| *P*[a] | | <0.0001 | NS | <0.0001 | <0.04 |
| (c) *Total* | | | | | |
| Breast cancer | 33 | 10.0 ± 1.0 | 4.6 ± 0.5 | 2.7 ± 0.3 | 1.0 ± 0.5 |
| Controls | 40 | 7.5 ± 0.6 | 5.1 ± 0.3 | 1.4 ± 0.1 | 1.5 ± 0.3 |
| *P*[a] | | <0.02 | NS | <0.0001 | <0.004 |

| | *n* | *Total lactogenic hormone (ng ml⁻¹)* BA | IRMA | BA/IRMA |
|---|---|---|---|---|
| (a) *Premenopausal* | | | | |
| Breast cancer | 7 | 17.2 ± 5.6 | 9.8 ± 4.7 | 2.2 ± 0.3 |
| Controls | 16 | 12.2 ± 1.7 | 8.4 ± 0.9 | 1.4 ± 0.1 |
| *P*[a] | | NS | NS | <0.005 |
| (b) *Postmenopausal* | | | | |
| Breast cancer | 26 | 11.5 ± 1.2 | 4.9 ± 0.5 | 3.0 ± 0.4 |
| Controls | 18 | 6.7 ± 0.8 | 5.4 ± 0.6 | 1.3 ± 0.1 |
| *P*[a] | | <0.0007 | NS | <0.0001 |
| (c) *Total* | | | | |
| Breast cancer | 33 | 12.7 ± 1.5 | 5.9 ± 1.1 | 2.8 ± 0.3 |
| Controls | 40 | 9.1 ± 0.9 | 6.7 ± 0.5 | 1.4 ± 0.1 |
| *P*[a] | | <0.009 | NS | <0.0001 |

[a]Mann Whitney U test: Breast cancer *vs* Control; NS = not significant.

**Figure 1** Basal prolactin and total lactogenic hormone levels (mean ± s.e.m.) by bioassay (BA) and immunoassay (IRMA) for breast cancer patients and controls. Mann Whitney U test: BA vs BA ○, $P<0.2$, ●, $P<0.0009$, RIA vs RIA $P=$ NS.

**Figure 2** Basal BA/IRMA prolactin ratio for patients with breast cancer (BC) and controls (CON) by menopausal status. Mann Whitney U test: BC vs CON: Total $P<0.0001$, Postmenopausal $P<0.0001$, Premenopausal $P=$ NS.

women, the elevation in the premenopausal subgroup failed to achieve statistical significance (Table II). Basal prolactin levels by microbioassay (BA) were slightly elevated over IRMA values in the control group but there was a significantly greater elevation in patients with breast cancer, reflected in a higher BA/IRMA ratio (Figure 2) which was significant in all subgroups except for premenopausal women. The elevation in BA/IRMA ratio was also significantly elevated for total lactogenic hormone activity (Figure 3).

### *Elevation of prolactin levels above upper limit of normal*

When a cut-off of the mean + 2s.d. was used for the upper limit of normal for prolactin values in the age-matched control groups, the number of breast cancer patients with elevated IRMA prolactin levels was of the same order as controls, reflecting the slightly skewed distribution of normal prolactin levels. However, basal BA prolactin levels were elevated in 18% of breast cancer patients (Table III). Relative prolactin bioactivity was found to be the best discriminator for basal levels with 61% of breast cancer patients having an elevated BA/IRMA prolactin ratio.

### *Correlation of prolactin levels with age*

As shown with a larger group of controls (Maddox et al., 1990), there was a weak inverse correlation of basal serum prolactin by BA and IRMA in normal subjects with age (Figure 4). However, breast cancer patients showed no correlation of basal BA or IRMA prolactin with age.

**Table III** Number of breast cancer patients and age-matched controls with basal serum prolactin levels by bioassay (BA) and immunoassay (IRMA) above the upper limit of normal

| | | *Number above upper limit (percentages in parentheses)* | | |
|---|---|---|---|---|
| | *n* | *BA*[a] | *IRMA*[b] | *BA/IRMA*[c] |
| Breast cancer | 33 | 6 (18) | 2 (6) | 20 (61) |
| Controls | 40 | 2 (5) | 1 (3) | 0 |

[a]Upper limit of normal basal BA prolactin: 15.2 ng $ml^{-1}$; [b]Upper limit of normal basal IRMA prolactin: 9.0 ng $ml^{-1}$; [c]Upper limit of normal basal BA/IRMA prolactin: 2.0.

### Discussion

This study has shown that in accordance with the observations of several authors (Franks et al., 1974; Sheth et al., 1975; Jones et al., 1977; Kwa et al., 1974), basal serum levels of prolactin in patients with breast cancer are generally within the normal range as determined by radioimmunoassay. Anderson et al. (1989) have reported no significant difference in the levels of basal lactogenic hormones between women with familial breast cancer and controls using both the radioimmunoassay and the original Nb2 bioassay. However, the number of patients studied was smaller and the two assays were not standardised to one reference preparation. Also the double-antibody radioimmunoassay was used (not the twin-site immunoradiometric assay) which may have influenced their results (Rose et al., 1988). The present study shows prolactin and total lactogenic hormone levels measured by microbioassay to be significantly elevated in breast cancer patients, compared with an age-matched control group. The majority of lactogenic activity in serum has been shown to be due to bioactive prolactin with only a minimal contribution from growth hormone. This finding is reflected in a markedly elevated basal BA/IRMA ratio for prolaction and total lactogenic hormone, with 61% of breast cancer patients having a basal BA/IRMA prolactin ratio above the upper limit of normal. The absolute levels of basal bioactive prolactin in breast cancer, however, are only moderately elevated above the normal physiological range. This may reflect a long-term modulator control of prolactin in the

**Figure 3** Basal BA/IRMA ratio for total lactogenic hormone (TLH) in patients with breast cancer (BC) and controls (CON) by menopausal status. Mann Whitney U test: BC *vs* CON: Total $P<0.0001$, Postmenopausal $P<0.0001$, Premenopausal $P<0.005$.

**Figure 4** Correlation of basal serum prolactin levels by bioassay (BA) with age for breast cancer patients and controls ($r$ = Pearson correlation coefficient).

breast, perhaps in concert with sex steroid hormones, leading to the induction and promotion of breast cancer.

The predominant prolactin moiety secreted by the pituitary is the monomer ('little' prolactin), which constitutes about 90% of total human pituitary prolactin extract; 'big' and 'big big' prolactin comprising 10–20% and 1–8% of the total, respectively (Garnier, 1978). There is evidence that the monomeric form may be more biologically active, measured by receptor binding activity, compared to the larger forms (Garnier *et al.*, 1978; Farkouh *et al.*, 1979). It may be that breast cancer patients secrete more of the monomeric form of prolactin, but serum factors may also play a significant role in the expression of biological activity of prolactin in peripheral blood.

This study has also demonstrated a moderate negative correlation between basal serum bioactive prolactin and age in healthy women, but no correlation was found for breast cancer patients. This is in agreement with the findings of Rose and Pruitt (1981) who attributed this lack of correlation in breast cancer patients to a relative and absolute hyperprolactinaemia throughout all age groups in women with breast cancer, thereby abolishing the normal negative correlation of prolactin within age.

Previous attempts to assess the importance of prolactin in breast cancer by immunoassay may have failed to measure the biologically important activity. However, whether this elevation in basal bioactive prolactin is involved in the aetiology of breast cancer by prolonged breast stimulation or is itself a consequence of the neoplastic process remains uncertain.

We are grateful to the National Institute of Diabetes, Digestive and Kidney Diseases (Baltimore, Md, USA) who kindly supplied us with the following NHPP materials as a gift to enable this research to be carried out: NHMDDAFP c11580, NIAMDDAFP 2312C2, NIAMDDAFP 97720133.

### References

ADDISON, G.M. & HALES, C.N. (1971). The immunoradiometric assay. In *Radioimmunoassay Methods*, Kirkham, K.E. & Hunter, W.M. (eds) Churchill Livingstone: Edinburgh, p. 447.

ALDINGER, K.A., SCHULTZ, P.N., BLUMENSCHEIN, G.R. & SAMAAN, N.A. (1978). Thyroid-stimulating hormone and prolactin levels in breast cancer. *Arch. Intern. Med.*, **138**, 1638.

ANDERSON, E., MORTEN, H., WANG, D.Y., BURNS, P., BIRCH, J. & HOWELL, A. (1989). Serum bioactive lactogenic hormone levels in women with familial breast cancer and their relatives. *Eur. J. Cancer Clin. Oncol.*, **25**, 1719.

BARNI, S., LISSONI, P., TANCINI, G. & 5 others (1986). Prolactin response to thyrotropin-releasing hormone in early and advanced human breast cancer. *Tumori*, **72**, 399.

FARKOUH, N.H., PACKER, M.G. & FRANTZ, A.G. (1979). Large molecular size prolactin with reduced receptor activity in human serum: high proportion in basal state and reduction after thyrotrophin-releasing hormone. *J. Clin. Endocrinol. Metab.*, **48**, 1026.

FRANKS, S., RALPHS, D.N.L., SEAGROATT, V. & JACOBS, H.S. (1974). Prolactin concentrations in patients with breast cancer. *Br. Med. J.*, **4**, 320.

GARNIER, P.E., AUBERT, M.L., KAPLAN, S.L. & GRUMBACH, M.M. (1978). Heterogeneity of pituitary and plasma prolactin in man: decreased affinity of 'big' prolactin in radioreceptor assay and evidence for its secretion. *J. Clin. Endocrinol. Metab.*, **47**, 1273.

JONES, M.K., RAMSAY, I.D., COLLINS, W.P. & DYER, G.I. (1977). The relationship of plasma prolactin to 17-B oestradiol in women with tumours of the breast. *Eur. J. Cancer*, **13**, 1109.

KWA, H.G., DEJONG-BAKKER, M., ENGELSMAN, E. & CLETON, F.J. (1974). Plasma prolactin in human breast cancer. *Lancet*, **i**, 433.

MADDOX, P.R., JONES, D.L. & MANSEL, R.E. (1989). A new microbioassay for the measurement of lactogenic hormones in human serum. *Horm. Res.*, **32**, 218.

MADDOX, P.R., JONES, D.L. & MANSEL, R.E. (1991). Basal prolactin and total lactogenic hormone levels by microbioassay and radioimmunoassay in normal human sera. *Acta Endocrinologica* (in press).

OHGO, S., KATO, Y., CHIHARA, K. & IMURA, H. (1976). Plasma prolactin responses to thyrotropin-releasing hormone in patients with breast cancer. *Cancer*, **37**, 1412.

ROSE, D.P. & PRUITT, B.T. (1981). Plasma prolactin levels in patients with breast cancer. *Cancer*, **48**, 2687.

ROSE, D.P., BERKE, B. & COHEN, L.A. (1988). Serum prolactin and growth hormone determined by radioimmunoassay and a two-site immunoradiometric assay: comparison with the Nb2 cell bioassay. *Horm. Metab. Res.*, **20**, 49.

SECRETO, G., RECCHIONE, C., CAVALLERI, A., MIRAGLIA, M. & DATE, V. (1983). Circulating levels of testosterone, 17B-oestradiol, luteinising hormone and prolactin in postmenopausal breast cancer patients. *Br. J. Cancer*, **47**, 269.

SHETH, N.A., RANADIVE, K.J., SURAIYA, J.N. & SHETH, A.R. (1975). Circulating levels of prolactin in human breast cancer. *Br. J. Cancer*, **32**, 160.

SINHA, Y.N., SELBY, F.W., LEWIS, U.J. & VANDERLAAN, W.P. (1973). A homologous radioimmunoassay for human prolactin. *J. Clin. Endocrinol. Metab.*, **36**, 509.